# Cytoplasm Types Affect DNA Methylation among Different Cytoplasmic Male Sterility Lines and Their Maintainer Line in Soybean (*Glycine max* L.)

**DOI:** 10.3390/plants9030385

**Published:** 2020-03-20

**Authors:** Chunjing Lin, Bao Peng, Yongkuan Li, Pengnian Wang, Guolong Zhao, Xiaoyang Ding, Rong Li, Limei Zhao, Chunbao Zhang

**Affiliations:** Soybean Research Institute, The National Engineering Research Center for Soybean, Jilin Academy of Agricultural Sciences, No. 1363, Shengtai St., Changchun 130000, China; lincj@cjaas.com (C.L.); pb@cjaas.com (B.P.); ykli@cjaas.com (Y.L.); pnwang@cjaas.com (P.W.); glzhao@cjaas.com (G.Z.); xyding@cjaas.com (X.D.); rli@cjaas.com (R.L.)

**Keywords:** Soybean, DNA methylation, cytoplasmic male sterility, whole-genome bisulfite sequencing, differentially methylated region (DMR)

## Abstract

Cytoplasmic male sterility (CMS) lines and their maintainer line have the same nucleus but different cytoplasm types. We used three soybean (*Glycine max* L.) CMS lines, JLCMS9A, JLCMSZ9A, and JLCMSPI9A, and their maintainer line, JLCMS9B, to explore whether methylation levels differed in their nuclei. Whole-genome bisulfite sequencing of these four lines was performed. The results show that the cytosine methylation level in the maintainer line was lower than in the CMS lines. Compared with JLCMS9B, the Gene Ontology (GO) enrichment analysis of DMR (differentially methylated region, DMR)-related genes of JLCMS9A revealed that their different 5-methylcytosine backgrounds were enriched in molecular function, whereas JLCMSZ9A and JLCMSPI9A were enriched in biological process and cellular component. The Kyoto Encyclopedia of Genes and Genome (KEGG) analysis of DMR-related genes and different methylated promoter regions in different cytosine contexts, hypomethylation or hypermethylation, showed that the numbers of DMR-related genes and promoter regions were clearly different. According to the DNA methylation and genetic distances separately, JLCMS9A clustered with JLCMS9B, and JLCMSPI9A with JLCMSZ9A. Thus, the effects of different cytoplasm types on DNA methylation were significantly different. This may be related to their genetic distances revealed by re-sequencing these lines. The detected DMR-related genes and pathways that are probably associated with CMS are also discussed.

## 1. Introduction

Instead of traditional sexual cross-breeding, the seed industry now primarily uses cytoplasmic male sterility (CMS) for hybrid seed production [1]. Because CMS lines generate pollen abortion, using a CMS system avoids the need to artificially remove the maternal line pollen in cross-breeding programs. CMS systems also improve the genetic purity of hybrid seeds and increase seed yield. A CMS system involves three-lines, a CMS line (A line), its maintainer line (B line), and a restoring line (R line) [2]. The A line is controlled by both nuclear and cytoplasmic genes and is the donor of the sterile genes in the cytoplasm. The B line is the donor of the fertility genes in the cytoplasm and nucleus. When a B line has been backcrossed with the corresponding A line for over 10 generations, the two lines share the same nucleus. To reproduce an A line, the A line (maternal line) is crossed to obtain fertile pollen from the B line (paternal line). Soybean (*Glycine max* L.) is a major crop that provides protein and oil. The first CMS systems were reported by Davis [3], but no further reported. The commercialized hybrid soybean line was developed using CMS system in 2002 [4], and different CMS types were discovered in the successions [5,6,7,8,9].

DNA methylation is an important regulatory mode in eukaryotes that plays major roles in maintaining genome stability and regulating gene expression. Most DNA methylation occurs as 5-methylcytosine [10]. The level of DNA methylation in a genome depends not only on the establishment and maintenance of DNA methylation (hypermethylation), but also on the loss of DNA methylation (hypomethylation). In general, hypomethylation induces gene expression and activates transposable elements, whereas hypermethylation reduces gene expression and inactivates transposable elements [11]. In plants and other organisms, DNA methylation occurs in three sequence contexts, CG (or CpG), CHG, and CHH (where H is A, T, or C) [12].

Differences in DNA methylation patterns between CMS lines and their maintainer lines have been reported in plants [13,14]. In wheat, the DNA methylation was significantly affected by CMS. Three alloplasmic-type A lines with different male sterile cytoplasms, ms (S) - 90–110 (S-type), ms (T) - 90–110 (T-type) and ms (K) -90–110 (K-type) were used to do methylation-sensitive amplified polymorphism (MSAP). K-type cytoplasm induced more differences than T-type or S-type cytoplasm, as was indicated by the ratios of methylated to fully methylated sites detected by MSAP. Because the K-type was between two genera (Aegilops and Triticum), whereas the T- and S-types were within Triticum genus between *Triticum spelta* and *Triticum timopheevii* species the genetic distance among the cytoplasm was greater among nucleus for the K-type than for the T- and S-types. [13]. These differences in genetic distance may explain the variations in methylation patterns. Although MSAP techniques have been used widely in plants, this method is not good at detecting cytosine methylation at non-CCGG sites [15]. Whole-genome bisulfite sequencing (WGBS) is a sensitive and stable method for characterizing genome-wide methylation patterns at single-base resolution and is the gold standard for detecting methylation at CG, CHG, and CHH sites [16,17]. Until now, there is only limited information available about whether DNA methylation is affected by different CMS types in hybrid soybean. Some reports have speculated that methylation levels, especially the methylation levels of some differentially methylated region (DMR)-related genes, are probably affected by CMS [18,19]. In this study, to analyze polymorphisms of DNA methylation and DNA methylation associated with CMS in soybean, we compared the cytoplasm types of three CMS lines (A lines) with the cytoplasm type of the maintainer line (B line) by WGBS. Compared with the shared maintainer line, the three alloplasmic types sterile lines are whether different in their genomic DNA methylation and what difference between them. At the same time, in order to understand what causing to the difference, we also re-sequenced the four lines, expecting to analyze the cytoplasm types affect DNA methylation reason.

## 2. Materials and Methods

### 2.1. Plant Materials

The plant materials used in this study were developed at the Jilin Academy of Agricultural Sciences (Changchun, China). The CMS RN-type was found in 1994 [20], followed by the CMS ZD- and XXT-types in 1998 [21]. In particular, the CMS line JLCMS9A (RN-type) was used as the female parent to produce the first commercialized hybrid HYBSOY-1 in the world in 2002 [22]. The soybean CMS lines (A lines), JLCMS9A, JLCMSPI9A, and JLCMSZ9A, have three types of sterile cytoplasm, RN-, ZD-, and XXT-types, respectively, and share the same maintainer line, JLCMS9B (B line). Seeds from the three sterile lines were developed over more than 15 generations by backcrosses with the maintainer line JLCMS9B as the male parent. For the present study, the CMS and maintainer lines were grown in the field. Intact young terminal leaves were harvested and immediately stored in liquid nitrogen for DNA extraction. Total genomic DNA was extracted using the cetyltrimethylammonium bromide method [23].

### 2.2. WGBS Library Construction

After agarose gel electrophoresis detecting no degradation, Qubit 2.0 fluorimeter (Life Technologies, CA, USA) was used to detect the concentration of the genomic DNA, and a NanoPhotometer^®^ spectrophotometer (Implen, CA, USA) was used to detect the purity. The genomic DNA was sonicated into 200–300-bp DNA fragments using a Covaris S220 ultrasonicator (Covaris, MA, USA). The ends of the fragmented DNA were repaired and poly (A) was added at the 3′ ends. All the cytosines had methylated DNA adaptors added. After bisulfite treatment using an EZ DNA Methylation Gold Kit (Zymo Research, Irvine, CA, USA), the unmethylated cytosines were converted to uracil, and then PCR amplified to thymine; while methylated cytosines remained after PCR. Finally, the DNA libraries were obtained. After constructing the libraries, initial quantification was calculated using Qubit 2.0, and each library was diluted to 1 ng/µL. The insertion length of the library was detected using the Agilent 2100 Bioanalyzer platform (Agilent, Santa Clara, CA, USA). The effective concentration of each library was quantified accurately by real-time quantitative PCR (Q-PCR; library effective concentration >2 nM) to ensure the quality of the library. The qualified libraries were sequenced by the Beijing Novo Zhiyuan Company (China) on an Illumina HiSeq Sequencing Platform (HiSeq PE125) using double-ended sequencing.

### 2.3. WGBS Data Analysis

After removing the adaptors, raw reads with an N ratio <10% (N represents undetermined bases), low quality bases (quantity score <20), and high proportion of low-quality bases (>50% of the whole read) were removed. The remaining reads were considered clean reads. Bismark software (https://www.bioinformatics.babraham.ac.uk/projects/bismark/) was used to compare the clean reads with the soybean reference genome sequence (https://phytozome.jgi.doe.gov/pz/portal.html#!info?alias=Org_Gmax). To identify methylation sites, the methylation level was calculated as ML = mC/(mC + umC) [24], where ML is the methylation level, and mC and umC are the numbers of methylated and unmethylated cytosines, respectively. To determine the influence of the bisulfite conversion rate, the methylation level was corrected as follows: ML_corrected = (ML−r)/(1−r), where ML_corrected is the corrected methylation level and r is the bisulfite non-conversion rate [25]. DMRs were identified using the Bioconductor package DSS [26,27]. The distribution and significance of the mapped DMR locations in the genome were analyzed using Circos maps [28]. During the methylation level analysis, the regions with the most differences in methylation levels between samples are generally selected for clustering and classification analyses [29]. Gene ontology (GO) functional enrichment analysis was performed using GOseq software [30,31], and Kyoto Encyclopedia of Genes and Genomes (KEGG) pathways were compared and analyzed [32]. The corrected data analysis was tested using *p*-values, and *p* < 0.05 was selected as the significance value. We also used the A lines and B line for clustering analysis during the methylation level analysis [33].

### 2.4. Re-Sequencing of the A Lines and the B Line

After detecting, method same as part of 2.2, DNA extracted from the soybean CMS lines (A lines) JLCMS9A, JLCMSPI9A, and JLCMSZ9A and their maintainer line JLCMS9B (B line) were randomly fragmented by sonication to 350 bp, then a Truseq Library Construction Kit (Illumina, TX, USA) was used to construct the libraries. The ends of the fragmented DNA were repaired, a poly(A) tail was added, sequencing adaptors were removed, then the fragments were purified and amplified by PCR. The libraries were sequenced on an Illumina Novaseq 6000 platform (Illumina, TX, USA). After constructing the libraries, the detecting method of the library is the same as part of 2.2. Clean data were obtained by filtering the raw data and the clean reads were aligned against the soybean reference genome (https://phytozome.jgi.doe.gov/pz/portal.html#!info?alias=Org_Gmax).

The re-sequencing data were analyzed to detect single nucleotide polymorphisms (SNPs) and insertions/deletions (Indels) (<50 bp) using MPILEUP in SAMTOOLS [34] and annotated using ANNOVAR [35]. SNPs can be used to calculate distances between different lines. A phylogenetic tree was constructed for the A lines and the B line using the neighbor joining method with 100 bootstrap replicates using TreeBeST 1.9.2 (http://treesoft.sourceforge.net/treebest.shtml). Methylation levels were calculated using a 2-Kb/bin sequencing environment, and a Pearson’s correlation coefficient analysis was performed [36].

## 3. Results

### 3.1. Whole DNA Methylation Levels of Soybean Lines with Different Cytoplasm types

We obtained 35.51–39.87 Gb of clean reads from the four libraries (three CMS A lines and the maintainer B line). The soybean genome is 1.1 Gb, so the sequencing depth was approximately 20×. The Q30 values were 94.89–96.97%, and the bisulfite conversion rate was over 99%. The unique mapping rate to the soybean reference genome was 65.27–74.53% (Table 1). These results confirm the WGBS data were enough to continue the analyses. The GC content of the JLCMS9A and JLCM9B was 24.14% and 23.23%, respectively. Whereas the GC content of the JLCMSPI9A and JLCMZ9A was lower, at 20.35% and 20.74%, respectively (Table 1).

In the four soybean lines, the methylated cytosine (mC) levels were 9.76–11.27%. Among the methylated cytosines, the methylated cytosine at CpG (mCpG) levels were 42.14–46.65%, methylated cytosine at CHG (H can be A, T, or G; mCHG) levels were 31.8–36.65%, and methylated cytosine at CHH (mCHH) levels were 1.91–3.29% (Table 2).

**Figure 1:**
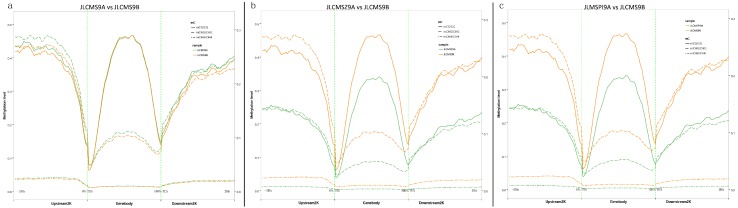
Distribution of methylated cytosines in the gene bodies and respective the 2-Kb upstream and 2-Kb downstream sequences among four soybean genomes. Different gene regions (X-axis), the upstream2K, genebodies, and downstream2K regions, were divided into 50 bins, and the methylation levels of all regions were respectively averaged. The methylation levels are shown on a CG sequence environment methylation level scale (right Y-axis coordinate) and on a non-CG sequence environment scale (left Y-axis coordinate). (**a**) JLCMS9A vs JLCMS9B; (**b**) JLCMSZ9A vs JLCMS9B; (**c**) JLCMS9A vs JLCMS9B.

**Figure 2:**
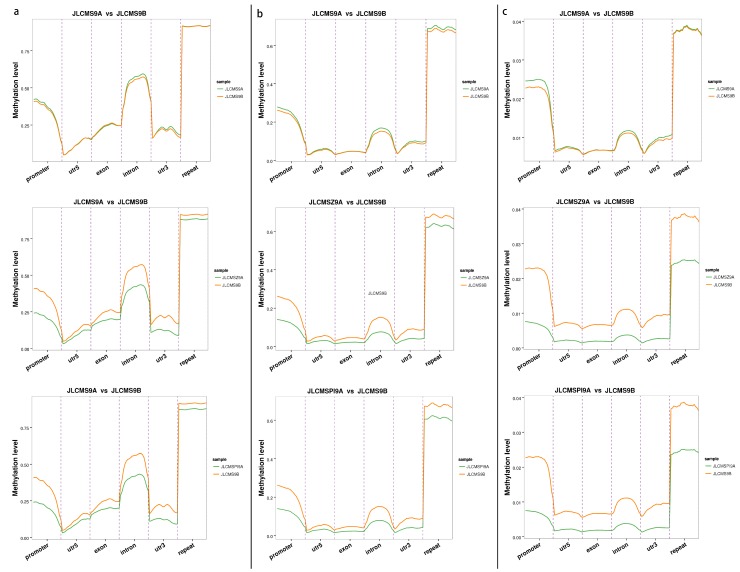
Distribution of methylated cytosines in the different genomic elements among four soybean genomes. The different genomic elements (X-axis), promoter, utr5 (5′-untranslatedregion), exon, intron, utr3 (3′-untranslatedregion), repeat, were divided into 20 bins, and then the methylation levels of all elements were respectively averaged. The methylation levels are shown on the Y-axis. The letter (**a**,**b**,**c**) represents sequence contexts mCG/CG, mCHG/CHG, and mCHH/CHH, respectively

We calculated the average cytosine methylation levels in the 2-Kb upstream sequences of the transcription initiation sites and 2-Kb downstream sequences of the transcription termination sites in each of the four genomes and the results are shown in Figure 1. Compared JLCMS9B among three sterile lines in the gene bodies, respective the 2-Kb upstream and 2-Kb downstream sequences the mCHH methylation level at less than 10% was lower than the mCG level or mCHG level. In gene body region, mCG methylation level was highest, at ~40–50% 50%, in any cytosine context for JLCMS9A. Additionally, in the gene bodies, respective the 2-Kb upstream and 2-Kb downstream sequences methylation patterns of JLCMSZ9A and JLCMSPI9A were very similar. The trends of the methylation in any cytosine context for both JLCMSZ9A and JLCMSPI9A were different from that of JLCMS9B. To better understand the DNA methylation levels of different functional genomic elements (promoters, exons, introns) and repeat sequences in each methylation context, cytosine-containing loci were located in different functional regions of the genome (Figure 2). Among them, intronic regions had the highest level of DNA methylation, followed by promoters, exons, and 5’ untranslated regions, and 3’ untranslated regions had the lowest level of DNA methylation in the CpG and CHG contexts. The methylation level curves for all cytosine contexts in the functional regions in the CMS lines almost coincided with those in JLCMS9B.

The correlation coefficients among the four lines were >0.50 (as shown in Figure S1). The results show that JLCMSZ9A and JLCMSPI9A, as well as JLCMS9A and JLCMS9B were closest, with correlation coefficients of >0.88 in the three mC contexts (Figure S1).

### 3.2. DMR Analyses in the Three CMS Types

**Figure 3:**
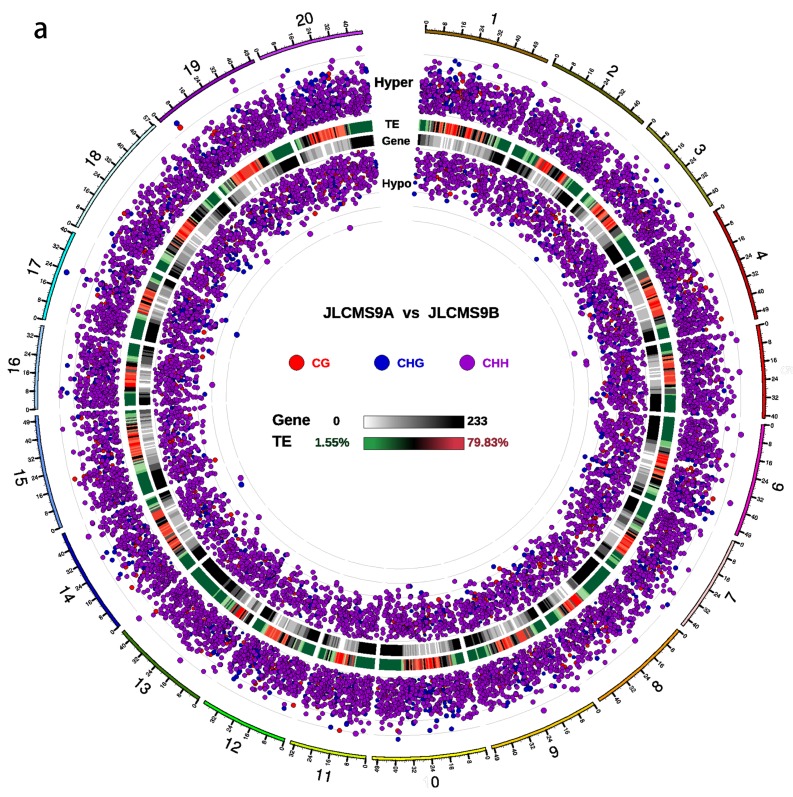

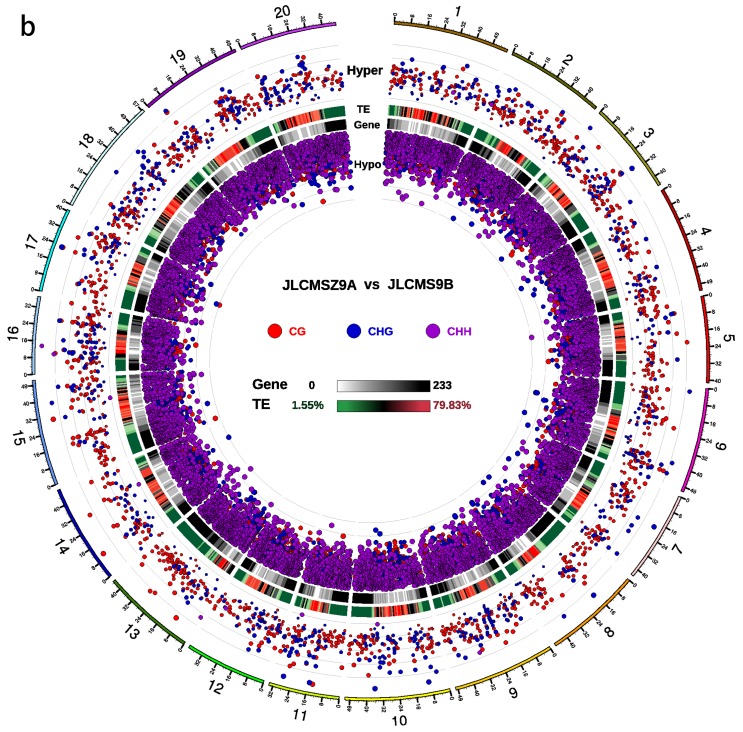

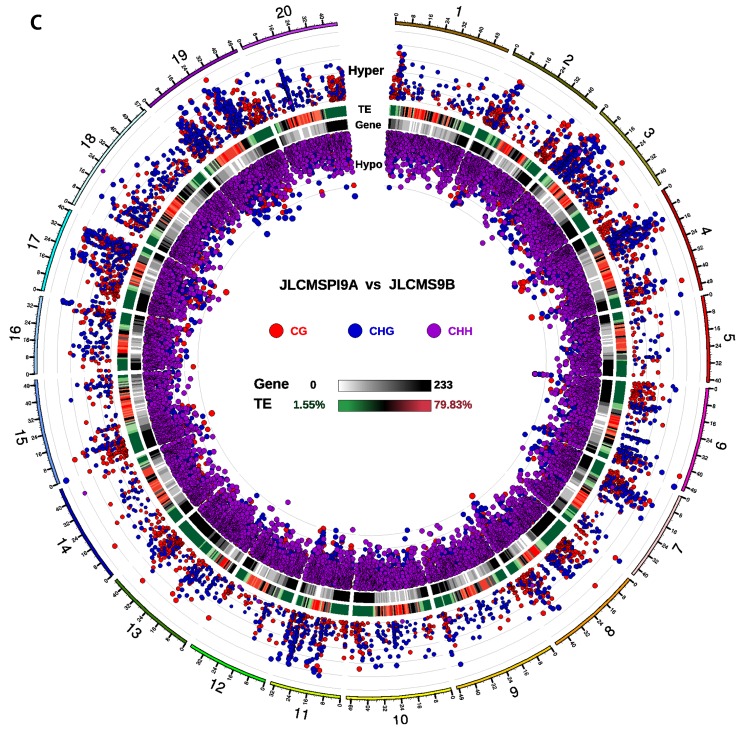
Cytosine methylation levels in three sequence contexts (CG, CHG and CHH) in differentially methylated regions (DMRs) as displayed by Circos maps. (**a**) JLCMS9A vs JLCMS9B; (**b**) JLCMSZ9A vs JLCMS9B; (**c**) JLCMS9A vs JLCMS9B. The red and blue dots denote hypermethylated and hypomethylated DMRs, respectively. The larger the dots, the greater the methylation differences between the two groups. From outside to inside, the circles are as follows: 1) Chromosome number and distance; 2) Hyper DMR log5 (|areaStat|), in the different positions the higher solid dots in the outward circle were more significan tdifference; 3) Heatmap of transposable elements (TEs) at chromosomal proportions (see color scale); 4) Gene density thermal map (Gene, see color scale), white to black indicates gene number from 0 to 233; and 5) Hypo DMR log5 (|areaStat|); in the different positions.

We used statistical significance values to reveal significant regional differences in the DMRs (Figure 3). Compared with JLCMS9B, the significant levels of hypermethylation or hypomethylation among the CHH sequences were higher than those among the CG and CHG sequences in the DMRs for JLCMS9A. Compared with JLCMS9B, the significant levels of hypermethylation among the CG and CHG sequences were higher than that of the CHH sequences in the DMRs for JLCMSZ9A and JLCMSPI9A.

**Figure 4:**
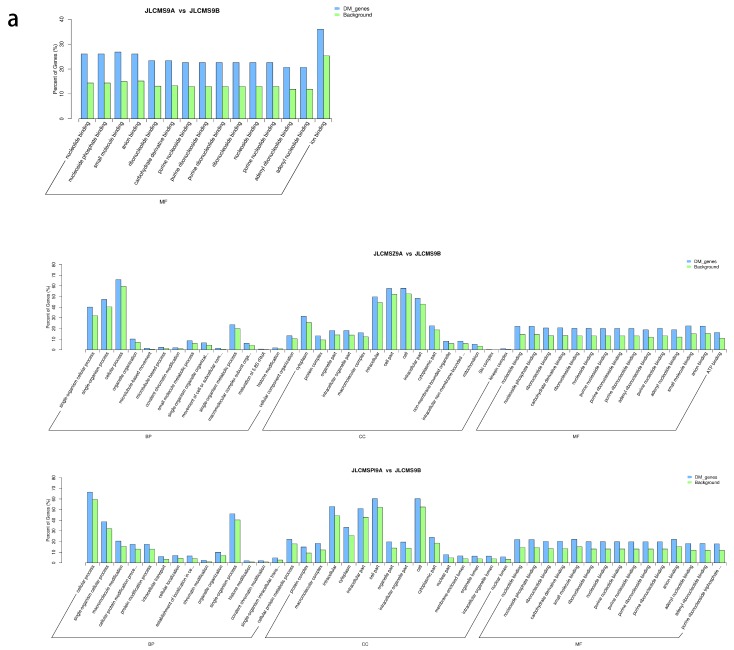

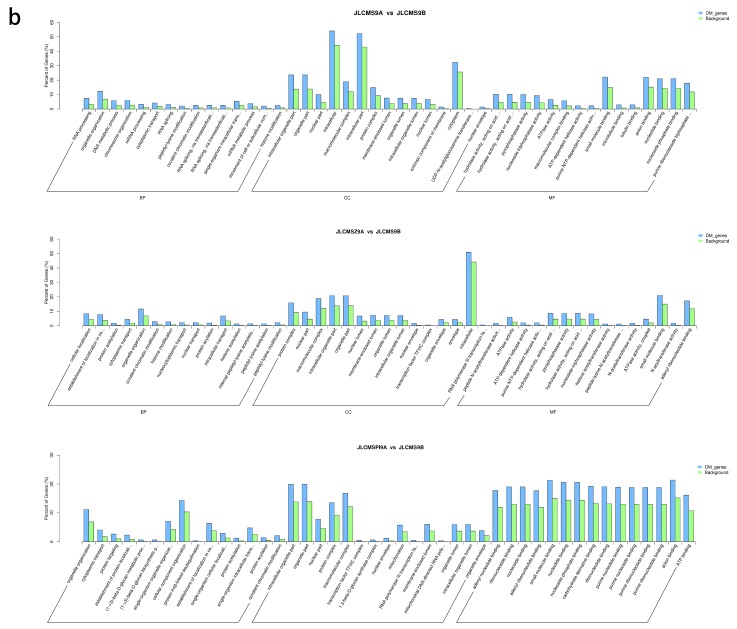

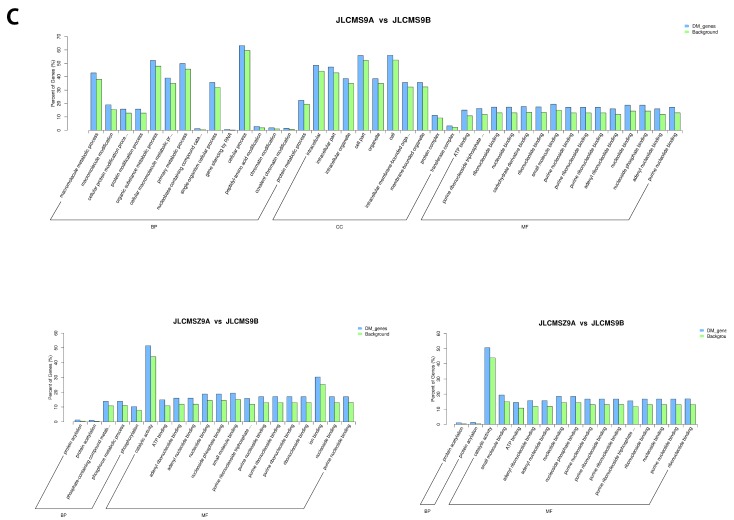
Gene ontology (GO) functional enrichment analysis in three sequence contexts of differential methylation regions (DMRs) in the whole soybean genome. Letter (**a**,**b**,**c**) represents sequence contexts mCG/CG, mCHG/CHG, and mCHH/CHH, respectively. BP: biological process; CC: cellular component; MF: molecular function.

The GO enrichment analysis of differentially methylated genes (DMGs) in JLCMS9A vs JLCMS9B in the CG background showed they were enriched in molecular function terms, while in JLCMSZ9A vs JLCMS9B and JLCMSPI9A vs JLCMS9B they also were enriched in biological process and cellular component terms compared with JLCMS9A (Figure 4).

**Figure 5:**
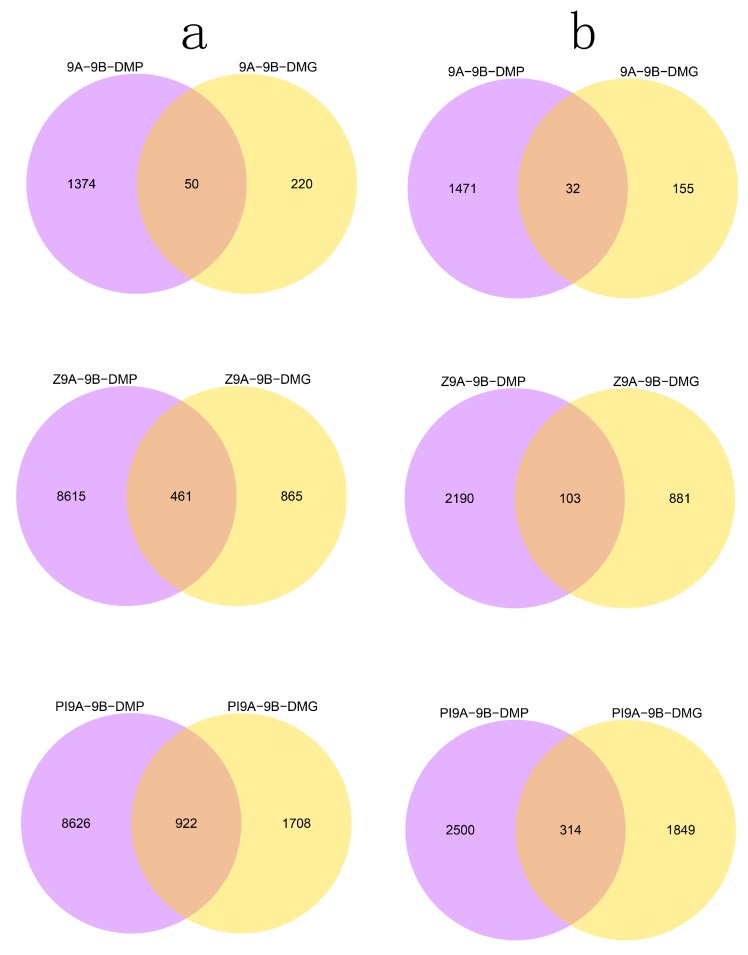

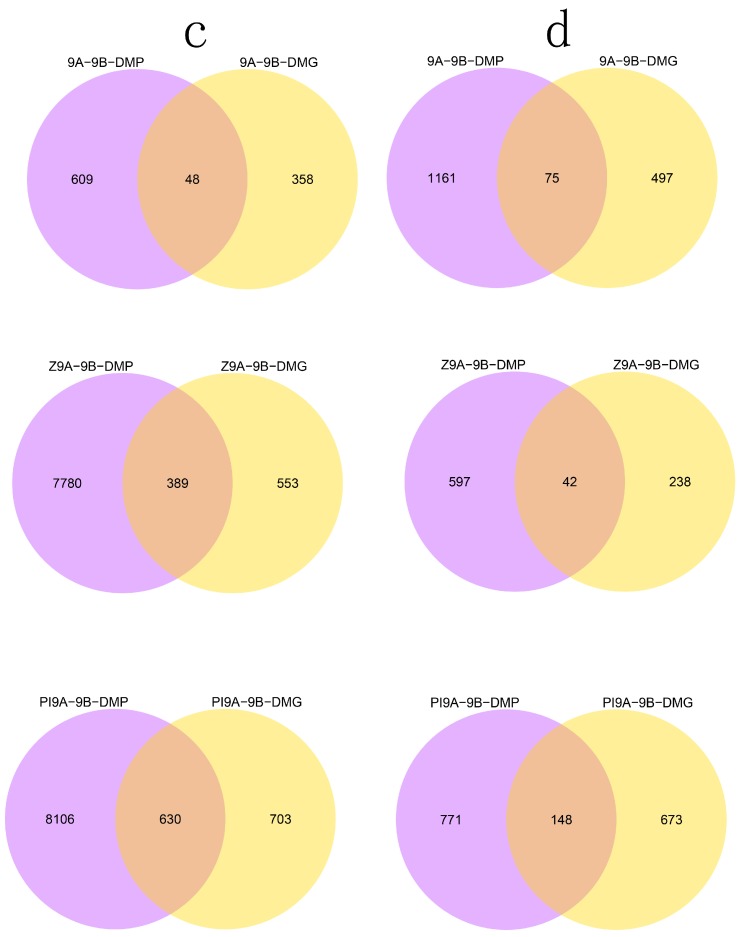

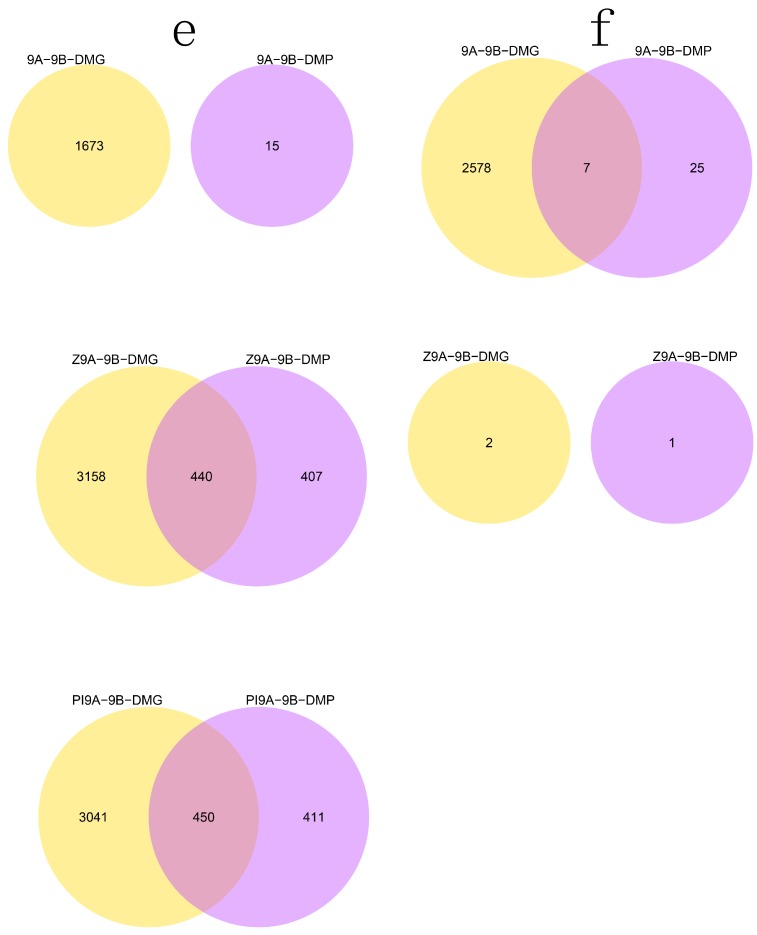
Venn diagrams of differentially methylated genes (DMGs) and differentially methylated promoters (DMPs) in the three sequence contexts of differentially methylated regions (DMRs) in the soybean genome. Yellow circles indicate hypomethylation; pink circles indicate hypermethylation. (**a**,**c**,**e**) hypermethylation in sequence contexts CG, CHG, and CHH, respectively. (**b**,**d**,**f**) hypomethylation in sequence contexts CG, CHG, and CHH, respectively. 9A-9B-DMG and 9A-9B-DMP, JLCMS9A vs JLCMS9B; Z9A-9B-DMG and Z9A-9B-DMP, JLCMSZ9A vs JLCMS9B; PI9A-9B-DMG and Z9A-9B-DMP, JLCMSPI9A vs JLCMS9B; DMG, differentially methylated genes; DMP, differentially methylated promoters.

A KEGG enrichment analysis of the DMGs showed that the enriched gene clusters in the CMS lines JLCMS9A, JLCMSZ9A, and JLCMSPI9A were similar to those in the maintainer line JLCMS9B. Compared with maintainer line JLCMS9B, the numbers of DMGs and differentially methylated promoters (DMPs) that were hypomethylated and hypermethylated among the three CMS lines were greatly different (Figure 5). Compared with JLCMS9B, the three CMS lines in the CG background had 1503–2814 hypermethylated DMPs and 187–2163 hypermethylated DMGs as well as 1424–9548 hypomethylated DMPs and 270–2630 hypomethylated DMGs; in the CHG background, they had 639–8736 hypermethylated DMPs and 280–1333 hypermethylated DMGs as well as 657–8169 hypomethylated DMPs and 406–942 hypomethylated DMGs; and, in the CHH background, they had 1–861 hypermethylated DMPs and 2–3491 hypermethylated DMGs, as well as 15–847 hypomethylated DMPs and 0–3598 hypomethylated DMGs.

### 3.3. DNA Methylation Levels and Genetic Distances between Three Soybean CMS Lines and the Maintainer Line

**Figure 6:**
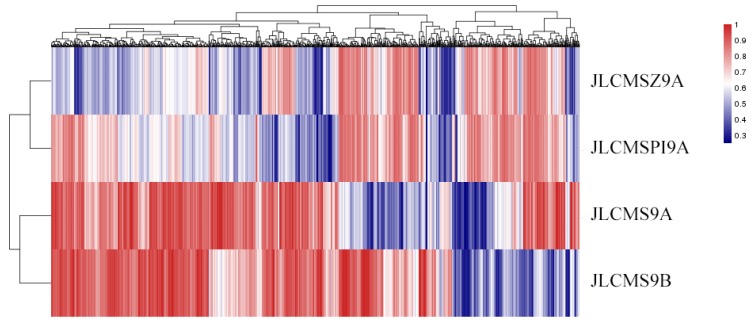
Epigenetic map of three cytoplasmic male sterility lines and the maintainer line according to differences in their methylation levels. Blue, low methylation level; red high methylation level.

We used the regions that had the most differences in methylation levels to construct an epigenetic map of the three CMS lines and the maintainer line (Figure 6). The results indicate that the pairs JLCMS9A and JLCMS9B, and JLCMSZ9A and JLCMSPI9A had short epigenetic distances and similar methylation patterns (Figure 6).

**Figure 7:**
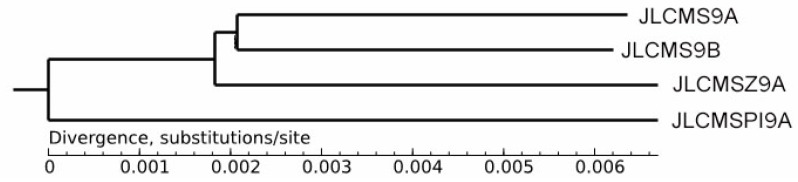
Phylogenetic tree of three cytoplasmic male sterility lines and the maintainer line constructed using the re-sequencing data.

We re-sequenced the genomes of the A lines and the B line and constructed a phylogenetic tree to determine their genetic distances (Tables S1 and S2). The results show that the pairs JLCMS9A and JLCMS9B and JLCMSZ9A and JLCMSPI9A shared close genetic distances (Figure 7). This finding is consistent with the results obtained from the epigenetic map (Figure 6).

### 3.4. DMRs Potentially Involved in CMS among the A Lines and B Line in Soybean

To determine if DMRs in the gene body or promoter regions affect the functions of genes related to CMS, we selected DMGs that had the highest statistical significance in the KEGG pathway enrichment analysis. The selected DMGs are listed in Table 3. We found that the DMGs that encode mitochondrial proteins or the related pathway were enriched in the three A lines and in the B line. For example, the genes encoding ATPase 4 and transcription factors such as WRKY42 and MYB2 were enriched between JLCMS9A and JLCMS9B as well as among JLCMSZ9A, JLCMSZ9A, and JLCMS9B.

## 4. Discussion

### 4.1. Cytoplasmic Effects of DNA Methylation among CMS Lines (A line) and Their Maintainer Line (B line)

As the A lines and the B line have the same nucleus donor, the most genetic differences were in the cytoplasm. We used the CMS lines JLCMS9A, JLCMSZ9A, and JLCMSPI9A to determine whether the cytoplasm type affected their nuclear methylation levels. Different genomic regions have different methylation patterns and perform different biological functions [37], so the differences in DNA methylation levels can help in understanding the roles of DNA methylation at the genome level [38]. We found significant differences in the DNA methylation levels in the cytoplasm of these three CMS lines. The RN-type cytoplasm donor is a landrace, Ru Nan Tian Er Dan [39], The ZD-type cytoplasm donor is cultivar line, ZD8319 [21], The XXT-type cytoplasm donor is not publisedpublished due to the need of patent application [21]. The cytoplasm ZD- and XXT-types in JLCMSPI9A and JLCMSZ9A, respectively, affected the nuclear methylation to a much greater extent than the RN-type in JLCMS9A. We re-sequenced the genomes of the A lines and B lines, which showed their genetic distances were very short, probably an effect of their cytoplasmic genomes. A similar result was also found in wheat [13]. In rice, the difference in the genetic distance between the cytoplasm and nucleus for the WA-type is much greater than for the G- and D-types because the former is between wild and cultivated rice species whereas the G- and D-types is within indica subspecies between African and Asian cultivars. [14]. The authors suggested that these differences may be responsible for the variations in methylation levels in rice genomes.

### 4.2. Enrichment Analysis of DMR-Related Genes (DMGs) Potentially Related to CMS 

CMS is most likely related to variations in the mitochondrial genomes of plants [18,40,41]. Five out of the six selected methylated genes in male sterile rice PA64S (sterility) were downregulated than PA64S (fertility), which indicated that DNA methylation is involved in the sterility–fertility transition of PA64S under two environmental conditions [40]. In soybean, genome-wide DNA methylation profiles of the CMS line NJCMS5A and its maintainer NJCMS5B have been reported, and several DMGs that participated in pollen and flower development were identified as possibly associated with CMS [18,41]. These results indicate that different cytoplasm types can affect nuclear DNA methylation levels and that DMRs may lead to sterility–fertility transitions. We also found mitochondrial DMGs and related pathways, including those involving transcription factors WRKY42 and MYB2, in JLCMSZ9A, JLCMSZ9A, and JLCMS9B. A mitochondrial DMG that encodes a WRKY transcription factor was reported recently in soybean [41]. But this gene ID is different to the mitochondrial DMG that encodes WRKY42 detected among the A lines and B line in this study. Despite these findings, the specific mechanism of sterility–fertility transition in soybean is still unclear and the effects of DMGs on sterility need further clarification. For example, further studies into the structure on buds [41] and RNA transcript expression in flower development [19] may help in understanding the relationship between DNA methylation and CMS in plants.

## 5. Conclusions

Different cytoplasmic affects on DNA methylation among different male sterile lines and their maintainer line in soybean using WGBS. Significant differences in DNA methylation levels and different methylation regions existed among three different male sterile cytoplasmic types. The ZD- and XXT-types of cytoplasm affected the methylation much more than the RN-type, and the genetic distances among these three cytoplasmic types probably lead nuclear methylation levels difference. The relationships among the four lines were the same as determined by both their genetic and epigenetic distances. Compared with JLCMS9B, the GO enrichment analysis of DMR- related genes of JLCMS9A revealed that their different 5-methylcytosine backgrounds were enriched in molecular function, whereas JLCMSZ9A and JLCMSPI9A were enriched in biological process and cellular component. The detected DMR-related genes and pathways that are probably associated with CMS also are discussed.

## Figures and Tables

**Table 1:** WGBS of soybean A-lines and B-line data summary and alignment statistics with reference genome.

Samples	Clean_Reads	Clean_Bases (Gb)	Q30 * (%)	Unique Mapping Rate (%)	GC Content ** (%)	BS *** Conversion Rate (%)
JLCMS9A	162450767	38.95	94.89	66.39	24.14	99.59
JLCMSZ9A	144302087	35.64	96.73	74.53	20.74	99.90
JLCMSPI9A	143848786	35.51	97.63	74.06	20.35	99.92
JLCM9B	162499642	39.87	96.97	65.27	23.23	99.54

* Q30 (%): The proportion of bases with Q value greater than 30 in all reads, Q30 means that the probability of base being tested error rate is 0.1%; ** GC Content (%): (G+C)/(A+T+G+C) × 100% in clean data; *** BS conversion rate (%): The ratio of C to T by bisulfite treatment.

**Table 2:** Proportion of the different types of methylated cytosine sites in four soybean genomes.

Samples	mC * Percent (%)	mCpG ** Percent (%)	mCHG *** Percent (%)	mCHH **** Percent (%)
JLCMS9A	11.27	46.36	35.66	3.29
JLCMSZ9A	10.16	45.77	35.88	1.91
JLCMSPI9A	10.48	46.65	36.75	2.08
JLCM9B	9.76	42.14	31.8	2.47

* mC means methylated cytosine; ** mCpG means methylated cytosine at CG site; *** mCHG means methylated cytosine at CHG site; **** mCHH means methylated cytosine at CHH site.

**Table 3:** Differentially methylated region (DMR)-related genes potentially involved in cytoplasmic male sterility and gene function among soybean A lines and B line.

Samples (A-Line vs B-line)	Gene ID	DMR Region	Type (Site/Level) *	Description
JLCMS9A/JLCM9B	GLYMA_09G056300	Exon	CHH/Hyper	Plasma membrane ATPase 4
JLCMS9A/JLCM9B	GLYMA_13G210600	Exon	CHH/Hypo	EFTM_ARATH Elongation factor Tu, mitochondrial
JLCMS9A/JLCM9B	GLYMA_20G050400	Exon	CHH/Hypo	MADS-box protein AGL80
JLCMSZ9A/JLCM9B	GLYMA_01G006400	Exon	CG/Hyper	Arginine--tRNA ligase, chloroplastic/mitochondrial
JLCMSZ9A/ JLCM9B	GLYMA_10G132200	Exon	CHH/Hypo	MYB2_ORYSJ Transcription factor MYB2
JLCMSZ9A/ JLCM9B	GLYMA_10G098600	Promoter	CG/Hypo	Uncharacterized mitochondrial protein
JLCMSZ9A/ JLCM9B	GLYMA_05G068000	Promoter	CHH/Hypo	Beta-amylase 3
JLCMSZ9A/ JLCM9B	GLYMA_10G111400	Promoter, exon	CG/Hyper	WRKY transcription factor 42
JLCMSZ9A/ JLCM9B	GLYMA_12G154400	Exon	CHG/Hyper	Cytochrome c oxidase
JLCMSPI9A/ JLCM9B	GLYMA_03G039900	Promoter, exon	CG/Hyper	Clustered mitochondria protein
JLCMSPI9A/ JLCM9B	GLYMA_03G087800	Promoter	CG/Hyper	WRKY transcription factor
JLCMSPI9A/ JLCM9B	GLYMA_07G105800	Exon	CG/Hyper	Pentatricopeptide repeat-containing protein
JLCMSPI9A/ JLCM9B	GLYMA_03G039900	Promoter, exon	CG/Hyper	Clustered mitochondria protein

* Hyper: Compared with B-line DNA methylation lever of cytosine increased in A-line; Hypo: Compared with B-line DNA methylation lever of cytosine decreased in A-line.

## Supplementary Materials

The following are available online at https://www.mdpi.com/2223-7747/9/3/385/s1.

## Abbreviations

CMS	Cytoplasmic male sterility
A line	Male sterile lines
B line	Maintainer line
WGBS	Whole-genome bisulfite sequencing
DMR	differentially methylated region
MSAP	methylation-sensitive amplified polymorphism
DMG	DMR-related gene
DMPs	differentially methylated promoter regions
mC	Methylated cytosine
umC	Unmethylated cytosine
Q-PCR	Real-time quantitative PCR
ML	methylation level
GO	Gene Ontology
KEGG	Kyoto Encyclopedia of Genes and Genomes.

## References

[1] Havey M. (2004). The use of Cytoplasmic Male Sterility for Hybrid-Seed Production. Molecular Biology and Biotechnology of Plant Organelles: Chloroplasts and Mitochondria.

[2] Zhang J., Sun H., Zhao L., Zhang C., Yan H., Peng B., Li W. (2018). Nectar secretion of RN-type cytoplasmic male sterility three lines in soybean [Glycine max (L.) Merr.]. J. Integr. Agric..

[3] Davis W.H. (1985). Route to hybrid soybean production. U.S. Patent.

[4] Zhao L., Sun H., Wang S., Wang Y., Hang M., Li J. (2004). Breeding of hybrid soybean HybSoy. Chin. J. Oil Crop Sci..

[5] Zhang L., Dai O., Huang Z. (1999). Selection of soybean male sterile line of nucleo-cytoplasmic interaction and its fertility. Sci. Agric. Sin..

[6] Gai J., Cui Z., Ji D., Ren Z., Ding D. (1995). A report on the nuclear cytoplasmic male sterility from a cross between two soybean cultivars. Soybean Genet. Newslett..

[7] Bai Y., Gai J. (2006). Development of a new cytoplasmic-nuclear male-sterile line of soybean and inheritance of its male-sterile restoration. Plant Breed.

[8] Zhao T., Gai J. (2006). Discovery of new male-sterile cytoplasm sources and development of a new cytoplasmic-nuclear male-sterile line NJCMS3A in soybean. Eupytica.

[9] Li J., Nadeem M., Sun G., Wang X., Qiu L. (2019). Male sterility in soybean: Occurrence, molecular basis and utilization. Plant Breeding.

[10] Vertino P.M., Yen R.W., Gao J., Baylin S.B. (1996). De novo methylation of CpG island sequences in human fibroblasts overexpressing DNA (cytosine-5-)-methyltransferase. Mol. Cell Biol..

[11] Deniz Ö., Frost J., Branco M.F., Migurl R.B. (2019). Regulation of transposable elements by DNA modifications. Nat. Rev. Genet..

[12] Jackson J.P., Lindroth A.M., Cao X., Jacobsen S.E. (2002). Control of CpNpG DNA methylation by the KRYPTONITE histone H3 methyltransferase. Nature.

[13] Ba Q., Zhang S., Niu N., Ma S., Wang J. (2014). Cytoplasmic effects on DNA methylation between male sterile lines and the maintainer in wheat (Triticum aestivum L.). Gene.

[14] Xu P., Yan W., He J., Li Y., Zhang H., Peng H., Wu X. (2013). DNA methylation affected by male sterile cytoplasm in rice (Oryza sativa L.). Mol. Breed..

[15] Fulneček J., Kovařík A. (2014). How to interpret methylation sensitive amplified polymorphism (MSAP) profiles. BMC Genet..

[16] Liu Y.B., Siejka-Zielińska P., Velikova G., Ying B., Fang Y., Marketa T., Bai C., Chen L., Schuster-Böckler B., Song C. (2019). Bisulfite-free direct detection of 5-methylcytosine and 5-hydroxymethylcytosine at base resolution. Nat. Biotechnol..

[17] Su Y., Bai X., Yang W., Wang W., Chen Z., Ma J., Ma T. (2018). Single-base-resolution methylomes of Populus euphratica reveal the association between DNA methylation and salt stress. Tree Genet. Genomes.

[18] Li Y., Ding X., Wang X., He T., Zhang H., Yang L., Wang T., Chen L., Gai J., Yang S. (2017). Genome-wide comparative analysis of DNA methylation between soybean cytoplasmic male-sterile line NJCMS5A and its maintainer NJCMS5B. BMC Genomics.

[19] Han S., Li Y., Li J., Zhang H., Ding X., He T., Ga J., Yang S. (2018). Genome-wide analysis of DNA methylation to identify genes and pathways associated with male sterility in soybean. Mol. Breed..

[20] Sun H., Zhao L., Huang M. (1994). Studies on cytoplasmic-nuclear male sterile soybean. Chin. Sci. Bull..

[21] Sun H., Zhao L., Huang M. (1998). ZA Type of cytoplasmic male-sterile breeding and primary research in soybean. Soybean Sci..

[22] Sun H., Zhao L., Huang M., Wang Y., Li J. (2003). Advances in utilization of heterosis in soybean. Chin. J. Oil Crop Sci..

[23] Beckmann J.S., Osborn T.C. (1992). Plant genomes: Methods for genetic and physical mapping. Plant Genomes: Methods for Genetic and Physical Mapping.

[24] Lister R., Pelizzola M., Dowen R.H., Hawkins R.D., Hon G., Tonti-Filippini J., Nery J.R., Lee L., Ye Z., Ngo Q.M. (2009). Human DNA methylomes at base resolution show widespread epigenomic differences. Nature.

[25] Xi Y., Li W. (2009). BSMAP: Whole genome bisulfite sequence MAPping program. BMC Bioinform..

[26] Krzywinski M.I., Schein J.E., Biro I., Connors J., Gascoyne R., Horsman D., Jones S.J., Marra M.A. (2009). Circos: An information aesthetic for comparative genomics. Genome Res..

[27] Wu H., Xu T., Feng H., Chen L., Li B., Yao B., Qin Z., Jin P., Conneely K.N. (2015). Detection of differentially methylated regions from whole-genome bisulfite sequencing data without replicates. Nucleic Acids Res..

[28] Park Y., Wu H. (2016). Differential methylation analysis for BS-seq data under general experimental design. Bioinformatics.

[29] Ujjwal M., Saurav M., Anirban M., Sanghamitra B. (2015). Analyzing large gene expression and methylation data profiles using StatBicRM: Statistical Biclustering-Based Rule Mining. PLoS ONE.

[30] Ashburner M., Ball C.A., Blake J., Blake J.A., Botstein D., Butler H., Cherry J.M., Davis A.P., Dolinski K., Dwight S.S. (2000). Gene Ontology: Tool for the unification of biology. Nat. Genet..

[31] Cozzetto D., Jones D.T. (2016). Computational Methods for Annotation Transfers from Sequence. Methods Mol. Biol..

[32] Minoru K., Susumu G. (2000). KEGG: Kyoto Encyclopedia of Genes and Genomes. Nuleic Acids Res..

[33] Smallwood S.A., Lee H.J., Angermueller C., Krueger F., Saadeh H., Peat J., Andrews S.R., Stegle O., Reik W., Kelsey G. (2014). Single-cell genome-wide bisulfite sequencing for assessing epigenetic heterogeneity. Nat. Methods.

[34] Li H., Handsaker B., Wysoke R.A., Fennel T., Ruan J., Homer N., Marth G., Abecasis G., Durbin R. (2009). The Sequence Alignment/Map format and SAMtools. Bioinformatics.

[35] Wang K., Li M., Hakonarson H. (2010). ANNOVAR: Functional annotation of genetic variants from high-throughput sequencing data. Nucleic Acids Res..

[36] Zhang Y. (2005). Quantitative analysis of the relationship of biology species using Pearson correlation coefficient. Comput. Eng. Appl..

[37] Cokus S.J., Feng S., Zhang X., Chen Z., Merriman B., Haudenschild C.D., Pradhan S., Nelson S.F., Pellegrini M., Jacobsenet S.E. (2008). Shotgun bisulphite sequencing of the Arabidopsis genome reveals DNA methylation patterning. Nature.

[38] Zhao X., Chai Y., Liu B. (2007). Epigenetic inheritance and variation of DNA methylation level and pattern in maize intra-specific hybrids. Plant Sci..

[39] Sun H., Zhao L., Huang M. (2001). Cytoplasmic-genetic male sterile soybean and method for producing hybrid soybean. U.S. Patent.

[40] Chen X., Hu J., Zhang H., Ding Y. (2014). DNA methylation changes in photoperiod-thermo-sensitive male sterile rice PA64S under two different conditions. Gene.

[41] Ding X., Wang X., Li Q., Yu L., Song Q., Gai J., Yang S. (2019). Metabolomics studies on cytoplasmic male sterility during flower bud development in soybean. Int. J. Mol. Sci..

