# Supplementary figures and images for: Cytoplasm Types Affect DNA Methylation among Different Cytoplasmic Male Sterility Lines and Their Maintainer Line in Soybean (Glycine max L.)
Source: Plants (Basel). 2020 Mar 20;9(3):385. doi: 10.3390/plants9030385 (PMC7155767; doi:10.3390/plants9030385)

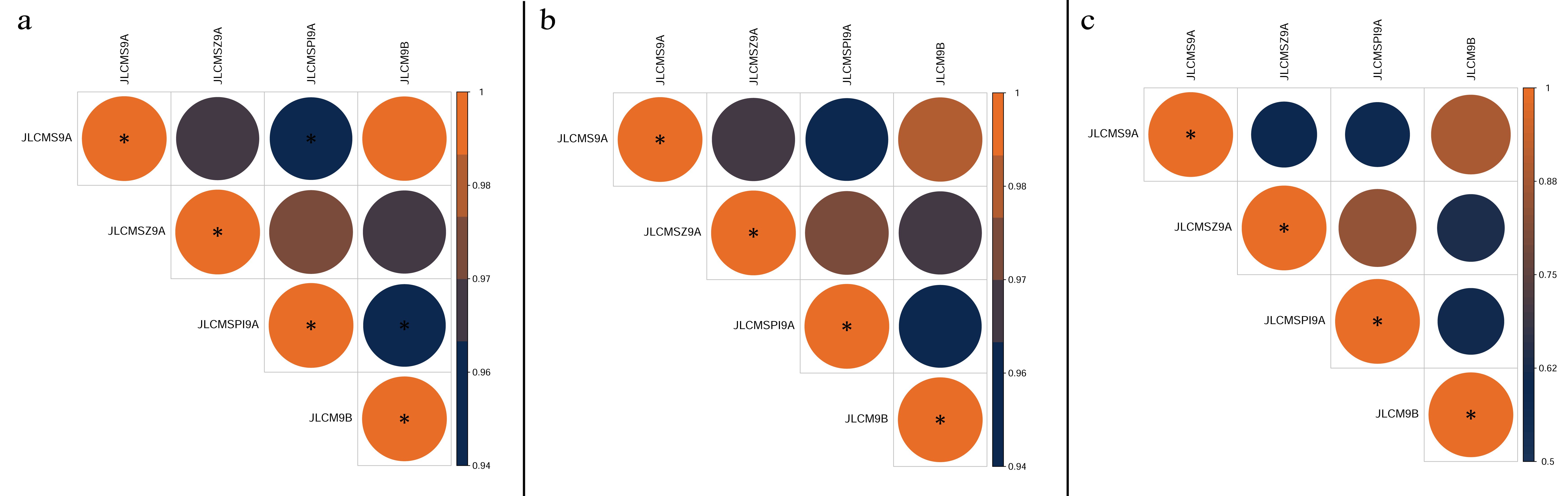

Supplement: Supplementary file 1 [file plants-09-00385-s001.zip › supplementary-729410/Fig S1.tif]
